# OPAL—The Toolbox for the Integration and Analysis of IoT in a Semantically Annotated Way

**DOI:** 10.3390/s21124002

**Published:** 2021-06-10

**Authors:** Philipp Hertweck, Tobias Hellmund, Jürgen Moßgraber

**Affiliations:** Fraunhofer IOSB, 76131 Karlsruhe, Germany; tobias.hellmund@iosb.fraunhofer.de (T.H.); juergen.mossgraber@iosb.fraunhofer.de (J.M.)

**Keywords:** Industrial Internet of Things, data integration, data processing platform, algorithm integration, SensorThings API, container technology, Docker, IoT platform

## Abstract

Industrial Internet of Things (IIoT) applications are being used more and more frequently. Data collected by various sensors can be used to provide innovative digital services supporting increasing efficiency or cost reduction. The implementation of such applications requires the integration and analysis of heterogeneous data coming from a broad variety of sensors. To support these steps, this paper introduces OPAL, a software toolbox consolidating several software components for the semantically annotated integration and analysis of IoT-data. Data storage is realized in a standardized and INSPIRE-compliant way utilizing the SensorThings API. Supporting a broad variety of use cases, OPAL provides several import adapters to access data sources with various protocols (e.g., the OPC UA protocol, which is often used in industrial environments). In addition, a unified management and execution environment, called PERMA, is introduced to allow the programming language independent integration of algorithms.

## 1. Introduction

The number of devices in the context of the Internet of Things (IoT) is (ever-)growing. In the year 2025 it is expected that more than 75 billion devices will be interconnected through the internet [1]. The omnipresent usage of IoT in all areas of everyday life is not surprising: personal tracking devices, environment observatories, smart home or smart city applications all try to make our life measurable and collect vast amounts of data. Next to these civil science driven applications, IoT plays a key role in the industrial area. Embedded computer devices supporting manufacturing processes—Industrial Automation and Control Systems (IACS)—are widely spread. Under the umbrella of the Industrial Internet of Things (IIoT), a growing number of devices and low power sensors transfer IoT applications in industrial domains. Additionally, the growing extent of wireless and mobile networks—especially the upcoming 5G mobile network—as well as specialized protocols for sensor connectivity, such as the Long Range Wide Area Network (LoRaWAN) [2], foster the usage of IoT devices.

Usually, the first step in the data analysis pipeline of sensor data is the collection and integration of various data sources, either from existing systems or from deployed sensors. Several standards address this use case, such as the Sensor Things API from the Open Geospatial Consortium (OGC) [3,4]. The next logical step in the analysis pipeline is the processing and analysis of the collected data: to take this step, we present OPAL, a lightweight and extensible toolbox covering the integration, management and processing of sensor data in a unified way. To ensure interoperability OPAL bases on open standards and semantic annotations.

In this paper, the following contributions are made:

(1) Nine high level requirements for an integrated data processing platform derived from several real world use cases are presented.
(2) A lightweight implementation—the OPAL toolbox—fulfilling these requirements is described.
(3) A new approach—called PERMA—is introduced. PERMA is a programming language independent platform for the integration of algorithms. PERMA internally uses the SensorThings API for the management of algorithms and their execution.

This paper is structured as follows: Use cases from different application domains, all covering aspects of the IIoT, are presented Section 2. These use cases are analysed to establish requirements for the OPAL toolbox. In Section 3 existing solutions are discussed. The subsequent sections present the the following aspects of OPAL: Section 4 describes the approach in detail, Section 5 the integration of data sources and Section 6 the processing steps. Finally, the OPAL toolbox is evaluated in Section 7 by proving the practical applicability through the implementation of the presented use cases.

## 2. Use Cases and Requirements

The results presented in this paper were developed during a project, covering several use cases from different application domains. These use cases were the basis for deriving the requirements, usually emerging during the implementation of IIoT applications. The presented OPAL toolbox focuses on the integration of heterogeneous data from several sources and the corresponding processing and analysis tooling.

### 2.1. Use Cases

For deriving the requirements and to develop the OPAL toolbox, the following five use cases in the context of IIoT have been selected and implemented:

UC1: activity detection
UC2: energy usage prediction
UC3: environmental monitoring
UC4: asset tracking
UC5: monitoring of food supply chains

- The first use case **UC1** comprises the integration of **activity detection**. In this context cameras record videos which were analyzed with an activity recognition algorithm; this algorithm detected time and place of the recorded activity. In industrial use cases, activity recognition is used for example to detect activities such as box lifting and so forth [5]. This analysis can be used for multiple purposes. For example prohibited actions, like people running in a factory hall can be detected. In the industrial context, it can also be used as a safety system: workers can easily call for help by waving their hands. This allows a surveillance system to automatically alert helpers. To fulfill privacy requirements, it is necessary to restrict the activity recognition to the site itself and to only save anonymous data. By only passing the analysis result (type of action, confidence level, position) it is ensured that no person is identifiable within the persisted data. The analysis result then can be integrated into a centralized system or situation view to visualize the information or to trigger further actions. In the context of this paper the focus is to identify popular places for specific activities, such as running.
- The second use case **UC2** is the **prediction of energy consumption**. In this use case, the energy consumption of a city district is measured and data is stored in a dedicated system used by public services. Due to its criticality and longevity, this system cannot be changed and extended easily. Therefore, it is hard to develop or evaluate new prediction algorithms that might be relevant for the users of the system. To overcome this issue, new algorithms should be integrated into an external system communicating with an interface of the system on site. This allows the development and evaluation of new energy prediction algorithms in an easier way.
- The third use case **UC3** deals with **environmental data**, especially water quality data from rivers, lakes and groundwater. In this case, the data is available in existing systems of German public authorities overseeing the condition of the environment. As described in UC2, it requires enormous effort to change and reuse these existing systems. To implement new analysis methods and to develop new applications the existing data needs to be available in a standardized way. Additionally, a data validation algorithm is implemented to check for measuring errors in the original data set.
- The fourth use case, **UC4**, deals with an **asset tracking system**. Based on ultra wide band (UWB) signals an indoor location system is established. With the help of four stationary transceivers an ad-hoc wireless network is realized. RTLSflares [6], the size of an USB stick, calculate the position based on the propagation time of the UWB signals. The resulting position is related to a local coordinate system, which depends on the position of the stationary senders. To ease out further integration of the position data and to allow the visualization on a map, those local coordinates need to be transformed into a world coordinate system. The OPAL toolbox supports the import of the assets position (local coordinates) as well as the execution of a coordinate transformation. Since the system requires a fine tuned calibration, optimization algorithms will be applied to determine the parameters of the coordinate transformation.
- In the final fifth use case, **UC5**, **food supply chains** are evaluated. While transporting perishable goods (for example meat), specific environmental conditions must be fulfilled all the time. To validate if these conditions were met, the foods condition is tested at the destination. A spectral analysis is done and the results are evaluated by a machine learning model to classify if the condition of the food is consistent with the stated environmental conditions during transportation. Overall, the OPAL toolbox should be able to handle heterogeneous data encodable in textual form, such as spectral data.

### 2.2. Requirements

The presented use cases form the basis for the OPAL toolbox. The following generalized technical requirements, split into data integration and processing, were derived from the use cases under consideration.

- **R1 Unified representation of sensor data**: All described use cases deal with time series data (uni- or multidimensional measurements at specific points in time). To reuse and share methods, know-how, data processing algorithms and visualizations the sensor data of the uses cases should be represented in a unified manner. In addition the unified representation can reduce complexity and simplify maintenance. However. Available semantic information, such as the unit of measurement needs to be preserved, since it might be crucial for the upcoming data processing and visualization.
- **R2 Seamless integration of sensor metadata**: as implied by R1, plain sensor data is not sufficient. To correctly process and reuse data, additional semantic metadata is needed. This comprises the storage and description of the measured phenomenon and its unit, time and place of the measurement or a description of the recording sensor. A tight coupling between sensor data and metadata is needed. This semantic information should not only be used for data integration but also throughout all data processing steps.
- **R3 Simple, but powerful data access**: Once the data is represented in a unified way, a simple data access mechanism needs to be established. This should be realized by a lightweight and easy usable protocol. Since data integration and processing is not limited to a single system, data exchange should be possible using common internet technologies. In this case Application Programming Interfaces (API) based on REST [7], over the Hypertext Transfer Protocol (HTTP) [8] are often used. To allow data exploration (e.g., for realizing new use cases), a single entry-point, referencing all available data is preferable. Additionally, a powerful query mechanism should be available offering query or search capabilities. These capabilities must not be limited to the sensor data itself, but must consider the respective metadata as well: spatial or temporal filtering should be supported just as string searches, and so forth.
- **R4 Interoperability**: Often, IIoT projects grow over time. In addition they are not isolated, but rather part of complex, long-living systems. Thus the number of involved components might increase over time. Furthermore, a technical or vendor lock-in should be avoided. Commonly accepted standards should be used, wherever possible. To ensure loose coupling of components (allowing the addition or removal of components without complex procedures), a messaging mechanism should be available to enable sensors sending their data proactively or enable the notification of specific components about a new event.

As described in the use case section, processing steps need to be integrated to realize the use cases. The main goal is to provide an approach, which allows us to focus on developing the processing algorithm, hiding the deployment and operational aspects. The following requirements aim at achieving this goal:

- **R5 External initiation**: To allow an easy integration into external systems and to increase the interoperability, it should be possible to trigger the algorithm through external systems. Knowledge about the execution environment should not be necessary. Therefore, a unified method is needed to allow either the execution by a user through a graphical interface, an external system or a specific event (e.g., new sensor measurement) to initiate and start processing.
- **R6 Parameterized processes**: To allow a more generic implementation of the processing steps, there should be a strict separation of the implementation and its configuration. This allows a flexible reuse of the algorithm. The external initiation should not only trigger the execution, but also pass the needed configuration to the algorithm.
- **R7 Execution environment agnostic**: To allow the reuse of implemented algorithms, its’ implementation should not depend on the physical execution environment. An appropriate abstraction layer needs to be established.
- **R8 Support for multiple programming languages**: It becomes apparent that depending on the use case different programming languages are used. Domain experts prefer different technologies—ranging from high level compiled languages like C++ or Java to interpreted languages like Python or even statistical languages like R [9].Therefore, the OPAL toolbox should allow a technology agnostic integration of processing algorithms to lower the bar regarding the use of the programming language of choice or the reuse of existing developments.
- **R9 Support of Machine Learning (ML) models**. Nowadays, machine learning plays a crucial role in processing and evaluating data. Furthermore, in the context of IIoT machine learning models are applied. Therefore, the execution of machine learning models (e.g., implemented with TensorFlow [10] should be explicitly foreseen in the OPAL toolbox.

## 3. Related Work

Research in IoT technology, as well as in the context of the Industrial Internet of Things gained a lot of attention in the last years. An overview of the technologies and challenges is given by Younan et al. [11]. Next to Cloud and fog computing, data fusion and machine learning are technologies for the future IoT. Those are also mentioned by Khan et al. [12]. Although several IIoT architectures and platforms have been developed [13,14], the following challenges have been identified by Khan et al.: efficient data management schemes, collaboration between heterogeneous IIoT systems and robust and flexible big data analytic technologies.

FIWARE [15] is a well-known IoT platform. It is built out of multiple components, called Generic Enablers. Those interface with IoT devices or third-party systems (e.g., HTTP [8], MQTT [16], JSON [17], OPC UA [18], provide data and API management as well as processing, analysis, and visualization components. FIWARE does not rely on a common data model. In contrast use case specific data models [19] are foreseen. For implementing the processing steps, different frameworks can be integrated.

In most of the IoT platforms there is no common data model available. This hinders the reuse and combination of data coming from different sensors. To overcome this shortcoming, a data integration step can be introduced. After that the data can be published leveraging web standards [20]. A possible standard to provide sensor measurements over the web is the Sensor Observation Service [21], standardized by the OGC. Its version 2.0 was released in 2012 and is based on Service Oriented Architecture (SOA) principles. Nowadays SOA interfaces are considered heavyweight. Therefore, the OPAL toolbox relies on the OGC SensorThings API [4], which uses the more lightweight REST paradigm. A detailed description of the SensorThings API can be found in the following Section 4.1. Instead of relying on one standard for integration and interoperability, sensor data can be semantically enriched [22]. Alternatively, Chaturvedi et al. suggested to introduce an interoperability layer to abstract from different data sources [23]. Jacoby et al. proposed to use semantic technologies to interchange IoT data between different platforms [24]. The Vital project is going a step further and provides a framework to access multiple IoT platforms by using semantic annotations [25].

There has not been much research in the integration of processing steps into IIoT based applications, yet. The processing steps are often considered as a separate layer in an IoT platform, without considering the integration and runtime aspects. One possibility to tackle the runtime aspect and to solve scalability issues is to use cloud resources [26,27,28]. Next to cloud-based solutions, distributed processing (Fog/Edge computing) is possible as well [29].

Commercial offerings for IoT platforms, especially from the cloud providers like Amazon AWS IoT [30], Microsoft Azure IoT [31] or Google Cloud IoT [32] are available, too. Their focus is on data acquisition and integration. Next to their IoT platforms, all of them offer additional cloud services. These are computing or storage solutions as well big-data processing frameworks or artificial intelligence (AI) methods. By combining multiple of those offerings, powerful and scalable applications can be developed. However, detailed knowledge about the cloud is needed, which might increase the barrier to enter. Interoperability with other solutions is not in the focus and therefore there is the risk of a vendor lock-in. In addition, such platforms cannot be used if there is the requirement to run applications in a local data center on-the premises.

Driven by technical progress, there are other approaches for algorithm integration. For example, using Function as a Services (FaaS) or Platform as a service (PaaS) solutions, processing pipelines can be easily implemented. In contrast to the OPAL toolbox they especially miss a high level concept for specifying and configuring the algorithm execution. Nevertheless, FaaS and PaaS solution can be integrated in the PERMA approach as well.

## 4. The OPAL Toolbox

In this section, we present OPAL—an open toolbox for the integration and analysis of time series data. This toolbox forms a lightweight platform for the integration of sensor data with the ability to flexibly integrate processing steps for this kind of data.

In contrast to existing IIoT platforms the focus of OPAL is on openness and interoperability. This is reflected in the requirements stated in Section 2. OPAL supports to flexibly integrate existing tools as well as to embed OPAL itself into existing systems. Due to its modality, different components of the toolbox can be selected for realization, depending on the use case. OPAL does not require any special hardware or infrastructure. Therefore, the components can be easily used in all kinds of environments ranging from single-board computers (like a Raspberry Pi) over self hosted data centers to cloud infrastructures. Due to these low requirements, OPAL is a lightweight platform, which is applicable to smaller use cases (like those mentioned in Section 2.1), too.

The main component is the data storage which supports the integration of heterogeneous data into a common data model. The novelty of the OPAL approach in this aspect is the single-point-of-contact for both sensor data, as well as processing steps. In contrast to proprietary solutions, the data model as well as the interface, are based on the SensorThings API. The standard is described in detail in the next Section 4.1. This centralized, and especially standardized, data representation allows the straightforward integration of existing applications into the OPAL toolbox. In addition, such a centralized data storage can break up data silos. Since data from various domains and use cases are represented in a common data format and made accessible in a unified way, applications for cross-domain use cases can be realized in a breeze.

**Figure 1:**
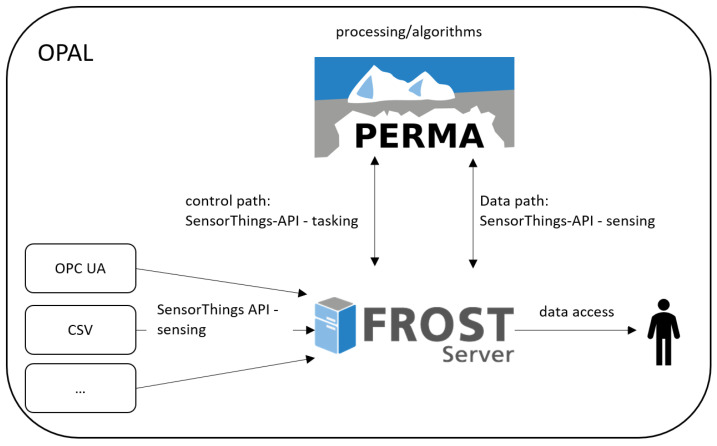
The OPAL toolbox.

The overall approach is visualized in Figure 1. The data for the different use cases is imported from various sources for example, CSV files, existing databases, external web services or by domain specific protocols like OPC UA. Part of the OPAL toolbox are adapters to collect data from various sources and to transform it into the SensorThings API data model. The available import adapters are described in detail in Section 5.2. As indicated in the figure, the OPAL toolbox relies on FROST [33], an open source implementation of the SensorThings API.

The second part of the OPAL toolbox deals with the deployment and integration of processing steps. It is realized trough a component called PERMA. As shown in Figure 1, both the control path (triggering and configuring the execution of the processing steps) and the data path (requesting the data to process as well as storing the processing results) are realized with the SensorThings API. This allows the establishment of FROST as the single source of truth—for data, as well as for analysis steps. PERMA and the integration of algorithms are described in detail in Section 6.

Since both, the raw sensor measurements as well as the processing results are stored in FROST, external systems or visualization applications can access all available data through the standardized SensorThings API. This simplifies the development and allows the reuse of existing tools.

### 4.1. SensorThings API

**Figure 2:**
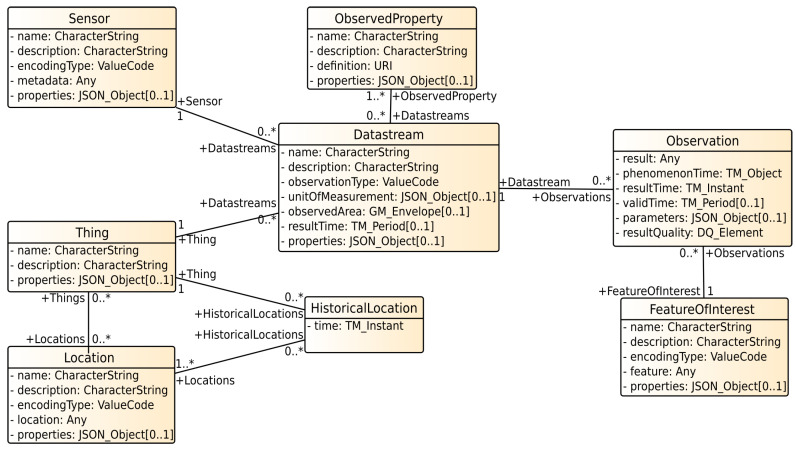
The SensorThings API data model [4].

The SensorThings API is a standard, developed by the Open Geospatial Consortium [3] in 2015. Its goal is to support the modelling, storage and exchange of sensor data. Therefore, it is destined to be used in the context of Industrial Internet of Things. The standard focuses on time-series data (measurements of the same observed property, for instance the temperature, taken at different points in time), which often occurs in IIoT use cases. Nevertheless, its data model allows the integration of other typed data as well. Next to the data representation itself, the SensorThings API allows linking measurements with the corresponding sensor metadata, as well as annotating the data semantically. The data model of the SensorThings API (to be more precise: the “OGC SensorThings API-Part 1 Sensing”, which covers sensor data and metadata management) is shown in Figure 2. The second part of the standard (“OGC SensorThings API-Part 2 Tasking Core”) covers tasking. It follows the same principles as part 1, but provides an extension for the data model. Part 2 forms the basis for the integration of analysis methods and therefore will be described in Section 6.

Almost all entities in the data model are self-explanatory and simplify the use. The central entity is a *Thing*. It represents a physical or virtual object. This is usually the entity being observed (e.g., a factory building, a river or a measurement station). A *Location* can be described as geographic locations, encoded as points or areas or symbolic locations, like postal addresses. If a *Thing* changes its’ location, the previous locations are stored as *HistoricalLocations*. A *Sensor* represents the actual sensor which is measuring an *ObservedProperty*. Both are connected to one another via the *Datastream* entity. This allows us to model a sensor (e.g., a wind sensor) that measures multiple properties (e.g., wind speed and direction). A measurement is an instance of an *Observation*. It is related to a *Datastream* and therefore to the *Sensor* and the *ObservedProperty*. If the sensing device measures a distant property (e.g., a satellite monitoring the surface of the earth), this can be described by a *FeatureOfInterest*. A detailed description of the entities can be found in the corresponding document of the standard [4].

Besides the data model, the standard defines a data access and query interface to exchange sensor data. This interface is based on commonly used protocols, paradigms and standards. Since the data can be read using the HTTP protocol, a browser sufficiently offers first insights into the data set. Create, update and delete operations are performed using the REST paradigm. To query data, OData [34] filters are supported, which include geospatial support. Sensor data might change frequently, therefore a lightweight publish/subscribe mechanism based on the MQTT protocol is utilized, which allows us to get notifications about new or updated data.

The SensorThings API, with its simple data model and interface is well suited to integrate the data in the OPAL toolbox in an open and standardized way [35]. To underline this it is now accepted by the European Commission as an INSPIRE good practice to provide spatial information [36].

## 5. Data Integration

As previously stated in Section 4, our approach contains two parts: data integration and on top of it, the integration of processing algorithms. This paragraph focuses on the first. As in every IoT application data collection and integration is one of the big challenges. To overcome the variety of different data sources, data models and access protocols we suggest to implement a central data store. This offers the possibility to unify the interface for the upcoming processing steps. In addition, this forms the basis for combining multiple data sources in a processing step. For example, weather data coming from environmental monitoring (an extension of UC3) can support the accuracy of energy predictions (UC4).

Until now, performance or scalability issues have not been observed in practice, even in projects containing a large amount of sensor data. Fischer et al. [37] present their *Urban Data Platform Hamburg*, a smart city platform for the German city of Hamburg integrating several sensor data sources. They are successfully using the same data storage solution, suggested for the OPAL toolbox-FROST-, for data integration and have not observed any performance issues.

If a centralized architecture is not possible, for example, due to legal or technical constrains, the OPAL approach can also be used in a federated manner: first integration and processing steps can be implemented in multiple instances of the OPAL toolbox, deployed and if necessary operated by different organizations and in distributed locations. Subsequently the pre-processed data (aggregated, anonymized, ...) can be collected in a central place. This is for example realized in the described use case for activity detection (UC1). The activity recognition module processes the captured video streams. Like intended, there is no personal data transferred and stored centrally.

The following Section 5.1 describes why the SensorThings API fulfills the requirements stated in Section 2.2. In the upcoming Section 5.2 the import adapters of the OPAL toolbox are described, followed by a collection of further compatible tools.

### 5.1. The SensorThings API: A Standard for Data Integration

Like motivated in the previously, a standardize data model as well as an access and query interface is needed. For fulfilling the unified representation of sensor data R1, the SensorThings API Section 4.1 is a suitable choice. The data model is able to cover the use cases UC1 to UC5 and yet it is easy to understand. Integration of the sensor metadata (R2) and therefore adding semantic information is foreseen. Going a step further, it is even possible to access the sensor data using semantic queries [38].

Like already mentioned, the SensorThings API offers query capabilities, based on the OData standard, which allows a simple, but powerful data access (R3). By passing a filter expression to the *$filter*-query parameter, the data can be searched. For example, *Observations?$filter=result gt 5* will only return Observations, which have a result greater than 5. Besides the comparison operators, mathematical and string functions are supported. Filter expressions can be combined using the logical operators *or*, *and* and *not*. Since sensor data usually is bound to a location, geospatial functions (calculating intersections, distances, ...) are supported in filter-expressions.

Besides the *$filter*-parameter, projections (only selecting specific attributes of an entity) are supported using the *$select*-query parameter. Since the resulting data might be large, it can be accessed in chunks. This pagination is known from relational databases as well and can be controlled using *$top* (number of entries to be returned) and *$skip* (skipping this number of first entries) parameters. In practice the *$expand*-parameter is quite useful. By default, relating entities are returned as references indicated by their id. To obtain those entities without a separate request, it is possible to specify the entities, that should be embedded in the response, by using the *$expand*-parameter.

Interoperability R4 can be realized by relying on an open standard, which is the SensorThings API. Since the SensorThings API itself relies on widely spread standards like HTTP and OData the technical interoperability is supported.

### 5.2. Import Adapters

Importing data into the SensorThings API is one of the key challenges in implementing the use cases. Therefore, several adapters for data import are part of the OPAL toolbox: ChillImport, UPCUA2FROST and the SensorThingsImporter.

**ChillImport** [39] is a web based graphical import tool for data represented in tables. It supports users without deeper technical knowledge to import CSV or Excel files into the SensorThings API. After uploading a file or specifying a URL where the file should be downloaded, the user is guided to create a new import configuration (created configurations can be stored and reused). First, the columns of the files containing the measurement time and the values are selected. In the next step, these are mapped to a *Datastream* of the SensorThings API. If the data stream does not exist on the server, it can be created. After this configuration, ChillImport will create the observations, based on the table lines. If a URL for the file was provided, ChillImport can run the import regularly to easily import new available data.

OPC UA is a common protocol to exchange data in industrial use cases. To integrate these data sources, the OPAL toolbox provides an import tool called **OPCUA2FROST**. For every Datastream, that is annotated with a *NodeId*-value in the *properties* attribute, a subscription to the corresponding node of an OPC UA server is established. Every measurement that is received over this subscription is transformed into an Observation and uploaded to the SensorThings API server.

Due to the variety of data the corresponding landscape of data sources proves to be very dissimilar. Therefore, it is not possible to provide a complete set of import adapters. To overcome this, the **SensorThingsImporter** [40] is part of the OPAL toolbox. It is a flexible framework to implement data imports. Common tasks like downloading and parsing CSV files, mapping them to Datastreams and uploading them to the server are already implemented. Use case or data source specific aspects can be easily realized by implementing the available Java-Interfaces of the SensorThingsImporter. In contrast to ChillImport, this is a more powerful and extensible tool; yet, it is much more complex and requires a deeper technical understanding.

### 5.3. Compatible Software Tools

Since the SensorThings API is an open standard, there is a growing ecosystem of tools. They can be easily combined with the OPAL toolbox.

First of all there are multiple server implementations of the standard available. The OPAL toolbox integrates **FROST** [33]. It is the first complete open-source implementation of the “OGC SensorThings API Part 1: Sensing”. It is written in Java and can be deployed to a variety of infrastructures (e.g., container images or a Helm-Chart for the deployment in Kubernetes are available). Next to the implementation of the “Part 1: Sensing”, FROST also supports “Part 2: Tasking Core”, which is used for the integration of the algorithms (see Section 6). An alternative is **GOST** [41], written in the GO programming language.

Viewing the data on the server is simply possible by using a web browser. Manipulating entities requires additional software tools like *Postman* [42] or *curl* [43]. Using such generic tools requires a good understanding of the SensorThings API data model. To simplify the interaction with the server a GUI component, called **FROST-Manager** [44] is available. The manager allows us to read, create, update and delete entities.

The data model of the SensorThings API is usable in a generic way. Therefore, for every use case domain specific objects must be mapped to the SensorThings API. Usually this can only be achieved by a technological expert user. It is possible to develop such a mapping with **FISA** [45]. This tool provides a web based interface for non-expert users. Input forms are provided to create new entities. Labels and descriptions are specific to the use case domain, so that the user does not directly interact with the technical vocabulary of the SensorThings API.

To ease the integration of the SensorThings API into applications developed in the Java programming language, the **FROST-Client** [46] is available. It is a Java-Library for accessing and manipulating the sensor data through a programming interface. This is especially useful when implementing analysis steps, as described in the next section.

In nearly every Industrial Internet of Things use case a visualization of the available data is needed. To support this, several tools are available. The **Masterportal** [47] is a web based portal which can visualize data from various OGC standards (including WMS [48], WFS [49], as well as the SensorThings API). The project is especially supported by the German city Hamburg to support their Smart City activities. It is well suited to visualize distributed sensors and their data on a map.

**Grafana** [50] is well known for monitoring IT infrastructures and services. Since it also supports SensorThings API data sources it can be used to build dashboards for Industrial Internet of Things use cases. Next to this freely available open source tools, the SensorThings API is integrated in popular commercial software tools, such as the software **ArcGIS** [51] from Esri.

## 6. Processing

In the last section we introduced the integration of heterogeneous data, by importing the available sensor data into the SensorThings API.

After data integration, there is usually the need to clean, validate or perform preparation steps on the data [52]. In a subsequent step, the data are usually analyzed by computational methods and further algorithms. The OPAL toolbox comprises both raw data and their analysis results in the same central store, implementing the SensorThings API; this not only saves development time and fosters reuse, but also provides a pathway to process the available data in a unified and standardized way through all steps of the pipeline. As the previous Section 2 elaborated, operational aspects should be considered as well. Practice showed that software deployment and runtime environment should not be underestimated.

For the purpose of processing sensor data, frameworks like NodeRED [53] or Apache Spark [54] are already available. However, such frameworks or approaches often prescribe a specific programming language. The production grade operation of these tools is expensive and requires knowledge about the tools. This requires additional training for the developers and prevents the integration of existing implementations. These issues contradict the initially stated requirement: support for multiple programming languages R8. Especially, when relying on BigData or machine learning platforms like Hadoop [55] or Kubeflow [56] a programming language-agnostic infrastructure is required or previously developed algorithms must be rewritten or adapted (e.g., from the development environment) to the production environment.

To overcome this, the OPAL toolbox provides an easy to use method to integrate analysis steps. For further unification, this integration for processing data relies as well on the SensorThings API. The following Section 6.1 describes the "OGC SensorThings API Part 2 Tasking Core", providing the underlying data model and interface to interact with the execution of analysis steps. The following Section 6.3 introduces PERMA. It is part of the OPAL toolbox and allows the integration of processing steps, based on the SensorThings API.

### 6.1. SensorThings API Tasking

**Figure 3:**
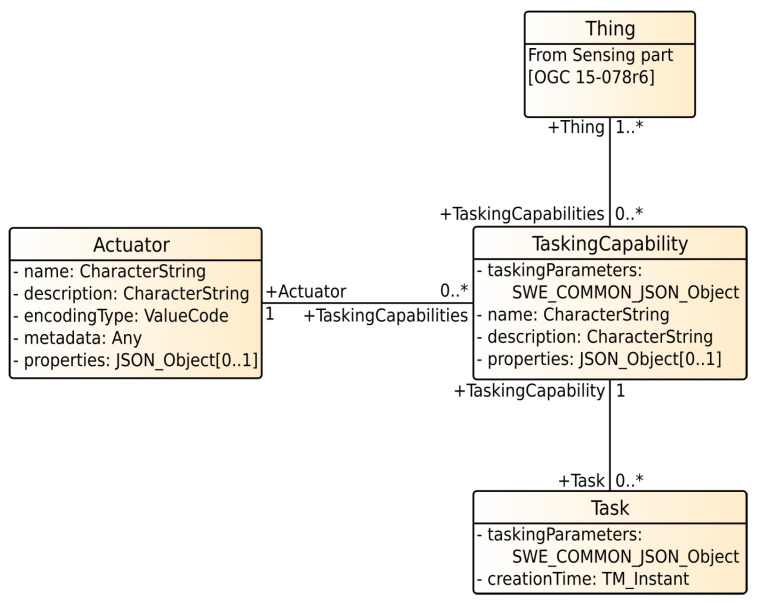
SensorThings API tasking data model [57].

Besides managing sensor measurements and metadata, the SensorThings API deals with actuators, controllable entities physically interacting with the real world [57]. This mainly extends the data model of the first part with new entities. These extensions are shown in Figure 3.

An *Actuator* in the context of the SensorThings API is a device that can be controlled. Its capabilities, which describe what an actuator can do, are represented as *TaskingCapability*. Next to a name and a description, a *TaskingCapability* contains *taskingParameters*. These formally describe the accepted parameters. The execution of a *TaskingCapability* is done by a *Task*. It is related to a *TaskingCapability* and contains the actual values for the *TaskingParameters*. Further semantics, for example, the current execution state of a Task, are not part of the SensorThings API. The connection in the data model between SensorThings API part one and two is realized by the *Thing*-entity, shown in Figure 2.

In the following, we consider an actuator which can open and close a window. Its *TaskingCapability* is to open or close a window and to ensure that the opening angle of the window matches a specific degree. Therefore, this capability has a *TaskingParameter* which describes that the *Actuator* can be controlled by passing a degree value. The actual execution (in this case opening the window) is realized as an instance of a *Task*: to open the windows for 50°, a *Task* with the *TaskingParameter* openPercentage=50° is created.

Analogous to the SensorThings API sensing part, entities can be accessed and manipulated via the same REST based interface. It should to be kept in mind that the SensorThings API only describes the data model and data access for controlling actuators. To actually execute a task, an actuator firstly needs to listen to a server, implementing the SensorThings API, either by querying via HTTP or by subscribing to the corresponding topic on the MQTT message bus. Secondly, the actuator needs to process the task definition, before it can actually interact with the physical world.

### 6.2. Using the SensorThings API for the Analysis of IoT Data

Apparently a physical IoT device, for example, a smart light bulb, a window opener or a robot with a paint nozzle can be considered as an Actuator of the SensorThings API. Nevertheless, this is not limited to hardware devices: virtual devices can be controlled by Tasks, too. Taking this idea a step further down the line, such a virtual device could have the ability to run an algorithm. Then, the execution of an algorithm can be interpreted as a *Task*. This allows us to formally express the degrees of freedom of the algorithm through *TaskingParameters* of the corresponding *TaskingCapability*. An actual execution of the algorithm is represented by the instance of a *Task* containing the values for the algorithms’ parameters.

### 6.3. PERMA

**Figure 4:**
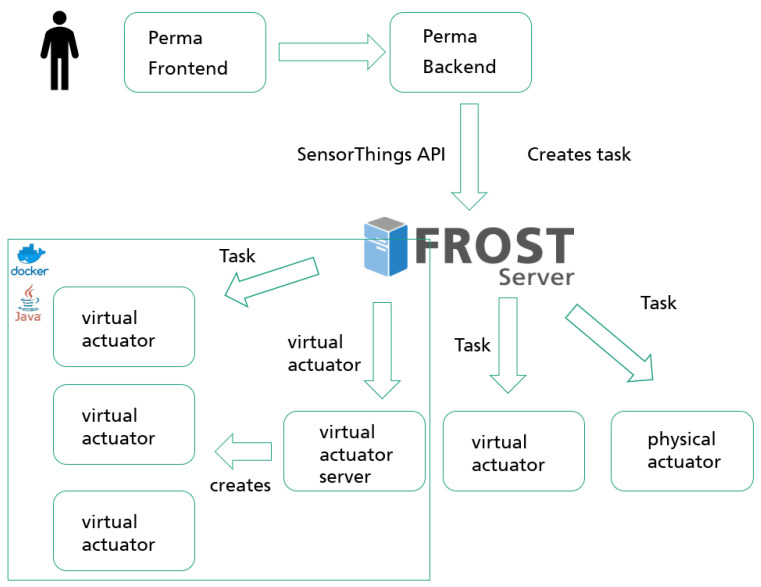
PERMA architecture.

The OPAL toolbox uses the previously described analogy for the integration of analysis algorithms. The execution component is called PERMA. Its overall architecture is shown in Figure 4.

Alike the data integration, FROST is used as an implementation of the SensorThings API as central component, too. PERMA itself consists of multiple modules. A web based front end is available for users to select the analysis algorithms, configure the parameters and to start the execution. This is supported by a server backend which creates the corresponding entities in FROST. The *Actuator*, which is able to execute the *Task* (running the processing algorithm), is in the context of this research now referred to as a *virtual actuator* and represented as an instance of an actuator in the SensorThings API data model. These virtual actuators are created by a component, called the *virtual actuator server*. This server is responsible to make sure a new instance of a virtual actuator is instantiated, once a new actuator entity is created in the SensorThings API. Those virtual actuators themselves subscribe to FROST and listen for newly created tasks (in this context also known as execution requests for the algorithms).

Keeping in mind the requirements described in Section 2, this approach offers the possibility to externally initiate and configure the processing execution.

To be able to run algorithms in a flexible and on-demand-manner a unified runtime, in which the virtual actuators can be executed, is needed. This runtime needs to be managed independently from the specific algorithms that are intended to be executed within this environment. This allows us to split the responsibility: a system administrator or platform operator manages the execution environment (analogous to Platform-as-a-Service-PaaS-offerings) while the algorithm can be implemented independently by domain experts. Due to the SensorThings API as the central tool for data linking and task execution, the data management is unified and the development of analysis algorithms is simplified.

To foster the integration of new algorithms or services, a mechanism to deploy new processing algorithms into the execution environment is required. As already mentioned, this is the responsibility of the server hosting the virtual actor. Internally, a virtual actuator server itself is represented as virtual actuator within the SensorThings API. It cannot execute an algorithm itself, but can deploy new virtual actuators which then can handle requests (more precise tasks for the algorithm execution). A virtual actuator server can have multiple *TaskingCapabilites* supporting the execution of different technologies (such as a Python-Script or JAR-file). In the context of this research we considered Java-based as well as a container-based [58] technologies. Therefore, a virtual actuator server in the context of the OPAL toolbox has two TaskingCapabilities: the first is starting a Java-based, whereas the second is starting a container-based virtual actuator.

### 6.4. Executing Algorithms as Virtual Actors

Implementing an analysis algorithm running in the Java environment requires to build a JAR file, containing a class which implements a simple interface, consisting of two methods:

- *getCapability()* which returns an instance of the SensorThings API entity TaskingCapability, describing the algorithm and its parameters.
- *handleTask(Task task)*, which accepts the Task with the parameters as argument. This is the place where the algorithm can be implemented.

This JAR file can be uploaded to PERMA using the web interface. When creating a new virtual actuator, the virtual actuator server will take the JAR file and extract the *TaskingCapability* by calling the appropriate method. Afterwards, a new *Actuator* and *TaskingCapability* will be created in FROST. In addition, a subscription is established to receive new tasks. If an analysis should be executed (realized by the creation of a *Task*) the virtual actuator server will call the *handleTask*-Method. Since the task itself is passed to the method, the implementation can easily access the parameters. Depending on the return value, the execution was successful or not.

In the OPAL toolbox it is foreseen that the implementation of the algorithms requests the needed data from the centralized server. As mentioned before, the analysis results should be fed back to the same instance. Anyhow, even if not foreseen it is technically possible to access external data sources as well.

To recap, the interaction flow can be summarized as:

1. A new algorithm is developed, either as a Java based application, implementing the given interface or by packaging it into a container.
2. In the PERMA web frontend a new virtual actuator is created. Next to the name of it, either a JAR-file is uploaded or the tag of a container image is provided.
3. Then, the virtual actuator server starts a new virtual actuator inside the execution environment. Furthermore, PERMA registers the actor and its TaskingCapability in FROST.
4. This newly created virtual actuator instance now subscribes to new tasks within FROST.
5. The execution of the algorithm can be started by creating a new task for the virtual actuator in the PERMA frontend. The algorithms parameters are passed as taskingParameters.
6. Through the task subscription, the virtual actor instance gets informed about the execution request. It queries the needed data, performs its calculations, writes the result back into FROST (specifically as sensor observations) and returns if its task was performed successful.

To integrate other programming languages and paradigms, a container-based (e.g., Docker [59] execution environment is foreseen. Instead of providing a JAR file, the algorithm implementation can be realized with an arbitrary language (machine learning models, for example, implemented with TensorFlow are possible as well), which is packaged with all required dependencies and a description file into a container image. The description file contains information about how to execute the application in the container and which parameters can be passed. Analogous to the java-based style, the deployment is achieved through the virtual actuator server.

Since FROST-Server is the single-point of interaction for the components in our data analysis pipeline, this approach is not bound to the PERMA front- and backend, but can be flexibly integrated into other systems.

This section described PERMA, an approach to use the SensorThings API tasking standard to integrate processing algorithms. This approach offers the possibility to decouple the implementation of algorithms from the execution environment. By providing the implementation either as JAR-file implementing a specific interface or as a container image, analysis steps can be easily brought to execution.

## 7. Evaluation

The evaluation of the OPAL toolbox is twofold. First, it is shown that the OPAL toolbox meets the stated requirements. This shows the fulfilment of the stated properties and quality attributes. Second, the realization of the use cases from Section 2.1 is described, which shows the applicability of our approach in those selected Industrial Internet of Things use cases. The use case applications themselves as well as the implementation of the data processing steps is out of the scope of this paper.

### 7.1. Realization of the Requirements

In this section, the previously mentioned requirements are recapped and it is shown how they can be met, using the OPAL toolbox.

**R1 Unified representation of sensor data**: A common representation is achieved by importing all data into the SensorThings API. A dedicated import step, implemented by the OPAL tools, reads data from the various available data sources, transforms it into the SensorThings API data model and writes the result to the centralized data store.

**R2 Seamless integration of sensor metadata**: The SensorThings API contains sensor measurements as well as sensor metadata. Relying on this standard ensures interconnected sensor data with its metadata offering the seamless integration semantic annotations.

**R3 Simple, but powerful data access**: The SensorThings API covers data access mechanisms. Relying on well known web technologies, such as HTTP and REST, accessing the data is very simple (to navigate through the data, a web browser is sufficient): since there are libraries for nearly every programming language supporting HTTP and REST, the chosen approach is beneficial. Implementing more advanced use cases, specific data can be requested by using ODATA queries. This allows filtering, as well as projections and limitations of the result set.

**R4 Interoperability**: Interoperability can be achieved by relying on accepted, widely used standards. Several widely used geospatial standards were developed by the Open Geospatial Consortium (OGC). Those are well accepted and widely used. The importance of the SensorThings APIs importance is growing. Therefore this supports the interoperability of the OPAL toolbox.

The following requirements (R5 – R9) cover the integration of the processing steps. These are met by the processing component PERMA inside the OPAL toolbox.

**R5 External initiation**: The processing steps can be triggered through the creation of a *Task* in the SensorThings API implementing server. There is no limitation, how this creation is realized. It can be done manually using the PERMA frontend, automatically in an already running virtual actuator in PERMA or by any other third-party system. Therefore, external initiation of the processing step is possible for every internet-bound device.

**R6 Parameterized processes**: Processing steps can be parameterized via the *TaskingParameters* of the *Task* that initiates the processing step. The available parameters, their data types and restrictions are formally specified through the TaskingCapability.

**R7 Execution environment agnostic**: The triggering of the processing step is independent from the execution environment. The creation of a *Task* in the SensorThings API hides the execution environment and is agnostic regarding the analysing tool, as well as its implementing technology and runtime environment. The algorithm is implemented environment agnostic, where possible. For simplicity, only an interface needs to be implemented in the Java case, while the registering and runtime aspects are hidden. For more flexibility the container approach can be used, in which the abstraction from runtime and execution aspects are pushed even further.

**R8 Support for multiple programming languages**: PERMA allows us to provide multiple execution environments, supporting different programming languages. For the implementation of the OPAL tools two environments for running processing steps were considered: first, a Java-based approach, which allows a simple implementation of processing steps. Second, a container-based environment which provides more flexibility for the implementation technology, but is more complex to use. While the first one is limited to the Java (or at least JVM based) programming language, the second one is programming language agnostic. Going further, the PERMA approach is not limited to those two execution environments. By implementing a new virtual actuator server an arbitrary technology can be easily integrated.

**R9 Explicit support for ML-models**: To support the evaluation of machine learning models, an integration into PERMA needs to be realized. In the OPAL, an ML algorithm has been both trained and utilized to prognose time series data. The application is based on Tensorflow [60] and containerized. This container can be executed using the PERMA container environment.

As shown, the OPAL toolbox in addition with the PERMA approach fulfills all the elaborated requirements.

### 7.2. Implementation of the Use Cases

Besides the fulfillment of the stated requirements the toolbox was evaluated by implementing real world use cases. The used components of the OPAL toolbox are summarized in Table 1.

For **UC1**, video cameras have been deployed. To fulfil the privacy requirements a processing unit was placed close to the cameras (edge processing). This unit executed **activity detection** on the incoming video stream. This was supported by PERMA: the detection algorithm, implemented as container executing a Tensorflow model (TaskingCapability) was executed on those units (virtual actuator server). Tasks were used to change the parameters of the activity detection. The detected events, together with the location and a timestamp were sent to the central SensorThings API server of the platform.

**Figure 5:**
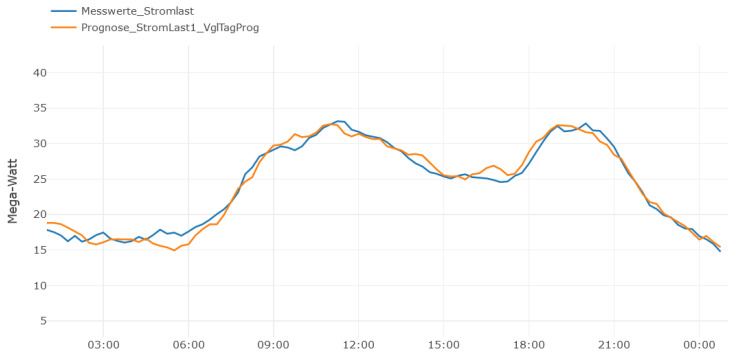
UC2 Energy prediction and actual usage.

The past energy usages of a district, used for **UC2** are available in an external system. Using the SensorThingsAPI importer, an import was developed copying the data to FROST. The **energy prediction** was developed in the Python programming language, using a common machine learning approach. The trained model and all required dependencies were written into a container image, which again was executed in the PERMA environment. The calculation was initiated by creating a Task, including the time range as parameter for the forecasted time period. Figure 5 visualizes the time series of the imported energy usage (blue line) as well as the prediction, executed for the same time period (orange line).

**Environmental data** is already collected by public institutions. In **UC3**, data from different sources were integrated. Since raw data might contain faulty values, a Java-based validation algorithm was developed. Using PERMA it is now possible to easily start a validation on the imported data.

*RTLS flares* measure the signal strength of reference senders in **UC4**. The measurement results were available as CSV files. For creating the initial entities and to upload the measurements, ChillImport was used. To allow **tracking a things location without GPS reception**, a coordinate transformation is needed. This transformation was developed as Python script. After the data import, PERMA was used trigger the transformation.

For **UC5** environmental parameters were measured during the transport. Additionally, a spectral analysis of the goods is executed afterwards trying to evaluate, if the transported good was in an environment that complies with the measured environment of the logistical chain. Using OPCUA2FROST, this data was imported through the OPC UA protocol into the SensorThings API. By triggering a Tensorflow neuronal network in PERMA, this spectral data in combination with the collected temperature is evaluated to decide if the measured condition fits with the current condition of the food.

## 8. Conclusions

In this paper OPAL was presented, a lightweight and flexible toolbox for integrating and processing sensor data. After establishing requirements derived from common use cases in the context of IIoT, an approach to integrate various data sources into the standardized SensorThings API was presented. This forms the basis for the second contribution: PERMA. An approach how to leverage the SensorThings API tasking part to integrate sensor data processing in a lightweight and flexible way.

Finally, the validation of the approach was executed. Firstly, by showing the fulfillment of all the stated requirements and secondly, the implementation of the IIoT uses-cases, presented at the beginning, practical applicability of the OPAL toolbox was described.

In future work it is planned to extend the PERMA approach, covering the data import aspects as well. Since PERMA allows the execution of an arbitrary algorithm, it is also possible to include the execution of the adapters. In contrast to the processing steps described in this paper, data import is a long running task. Logically emerging requirements, such as monitoring processes, and restarting/resuming these tasks need to be addressed in future work. Especially, for importing tasks, a declarative approach is under consideration.

Finally, especially when IIoT use cases should be implemented in a cloud or edge data center the topics of distributed algorithm execution and dealing with unreliable/changing IT infrastructure needs to be investigated. In addition, federated approaches, such as those mentioned in Section 5 could be considered in detail as well.

## Figures and Tables

**Table 1:** Summary of the realized use cases.

Use Case	Data Integration	Processing
activity detection	-	activity recognition (Tensorflow)
energy usage prediction	SensorThingsImporter	ML algorithm (Python)
environmental monitoring	SensorThingsImporter	data validation (Java)
asset tracking	ChillImport (CSV)	coord. transformation (Python)
food supply chains	OPCUA2FROST	neuronal network (Tensorflow)

## Data Availability

Not applicable.
